# Joint association of physical work demands and leg pain intensity for work limitations due to pain in senior workers: cross-sectional study

**DOI:** 10.1186/s12889-020-09860-6

**Published:** 2020-11-18

**Authors:** Sebastian Venge Skovlund, Rúni Bláfoss, Emil Sundstrup, Kristina Thomassen, Lars L. Andersen

**Affiliations:** 1 Musculoskeletal Disorders and Physical Workload, National Research Centre for the Working Environment, Copenhagen, Denmark; 2 Department of Sports Science and Clinical Biomechanics, Research Unit for Muscle Physiology and Biomechanics, University of Southern Denmark, Odense, Denmark; 3 Sport Sciences, Department of Health Science and Technology, Aalborg University, Aalborg, Denmark

**Keywords:** Manual labour, Musculoskeletal diseases, Work ability, Occupational medicine, Ergonomics, Workplace, Sustainable employment

## Abstract

**Background:**

Leg pain, especially of the knees and hips, is common among senior workers and may limit the ability to perform physically demanding work. In light of the aging workforce, this study determined the joint association of physical work demands and leg pain intensity for work-limiting pain in senior workers.

**Methods:**

Currently employed senior workers (≥50 years) participated in the SeniorWorkingLife study in 2018 (*n* = 12,879). Associations between the combination of physical work demands and leg pain intensity (interaction) with work-limiting pain (outcome) were modeled using binary logistic regression analyses while controlling for potential covariates.

**Results:**

We found a significant interaction (*P* < 0.001) between physical work demands and leg pain intensity for work-limiting pain. The combination of higher physical work demands and higher leg pain intensity had the worst outcome in terms of the odds of experiencing work-limiting pain. For example, 70% of those with the combination of high physical work demands and leg pain intensity ≥7 (scale 0–10) experienced that the pain limited them to at least some degree in their work.

**Conclusions:**

The combination of high physical work demands and high leg pain intensity are associated with limited ability to perform work among senior workers. These findings highlight the importance of prioritizing the physical work environment in physically demanding occupations, particularly among senior workers, for prolonging working life. Thus, adjusting the work demands, e.g. through use of assistive devices, and lowering the pain, e.g. through physical rehabilitation, may be necessary to sustain work ability to a high age in this group of workers.

**Trial registration:**

This was registered as a cohort study in ClinicalTrials.gov (Identifier: NCT03634410) on the 18th of August 2018 (Retrospectively registered).

## Background

Musculoskeletal disorders (MSD) in both upper and lower body are highly prevalent and debilitating worldwide, with upper body MSD such as low-back pain and neck-shoulder pain estimated to affect approximately 577 and 289 millions (~ 8 and 4% of the global population in 2017) worldwide, respectively, and lower body MSD in form of osteoarthritis of the hip and particularly the knee estimated to affect around 300 millions wordwide (4% of the global population in 2017) [1, 2]. However, recent research reviews indicate that knee−/hip osteoarthritis and other lower body MSD have received less attention in research in work environmental rehabilitation than low-back and neck-shoulder pain [3]. The reason for this may be that lower body MSD is less prevalent in the working population as a whole, and therefore largely overseen as a problem in specific target groups such as older [2, 4] and female workers [4–6], who are generally more affected by MSDs like low-back pain and osteoarthritis.

MSD are associated with high personal costs such as negatively affecting the ability to work [7–10] and quality of life [11]. Also, MSD may affect employers and society through increased risk of sickness absence [9, 12–16] and increased risk of premature exit from the labour market [17–19]. A recent survey suggested that approximately 28% of Danish senior workers aged 50–64 years feel limited in their job due to MSD, with even higher prevalences among senior workers in manual occupations [20]. Self-reported work limitation due to musculoskeletal pain has previously been associated with a 54% increased risk of long-term sickness absence [16], emphasizing the relation between MSDs, health, and work.

In Denmark, a relatively large proportion of the working population holds manual jobs that are physically demanding (depending on definitions) [21]. According to a large-scale study among the general working population in Denmark, approximately 40% of Danish workers report to walk or stand for at least ¾ of their workday, while approximately 17% report to be squatting or kneeling at least ¼ of their workday [22]. While the etiology of MSD is multifactorial [23], the working environment plays a huge role in the occurrence and retention of MSD, with physically demanding manual labour being associated with an increased risk of MSD [24, 25]. Knowing that physical capacity decreases inherently with age [26], some senior workers may not have the capacity to overcome the physical demands at work. In combination with the increased prevalence of MSD with age [21, 27], this could lower work ability [28–30] and increase the risk of leaving the labour market prematurely. Due to the demographical developments, the number of older workers is gradually increasing [31], and determining factors that could mediate the practical consequences of MSD may provide useful knowledge for the practitioners at the workplace to act on in the pursuit of sustainable employment [32].

Importantly, many workers have MSD without reporting lower work ability and increased sickness absence [29, 33], which may be explained by – amongst others – factors as musculoskeletal pain intensity and location, physical work demands, age, and sex [8–10, 14, 29, 34–40]. For example, it is possible that a senior employee with MSD may be capable of going to work and take full care of his/her work duties if it is a sedentary office job, whereas another senior employee with MSD may not be able to perform his or her job tasks if it is a job that is physically demanding. Also, women may be predisposed to work limitations compared to men as they typically possess lower muscle mass and muscle strength [41] and thus lower physical capacity to compensate with when dealing with MSD. Furthermore, a study among healthcare workers showed that the pain thresholds associated with increased long-term sickness absence differed between pain locations, with knee pain being associated with increased long-term sickness absence at the lowest pain threshold (i.e. ≥3 on a scale from 0 to 9) compared to low-back and neck−/shoulder pain (5 and 4, respectively) [14], potentially indicating a relatively larger impact of knee pain on working functional capacity compared to low-back and neck−/shoulder MSDs. This study and others [8, 9, 40] underline the importance of pain intensity on the degree of functional limitation, with higher pain intensities being associated with higher risks of work disability and sickness absence.

In this confirmatory cross-sectional study among senior workers, we aim to investigate the joint associations of physical work demands and leg pain intensity with work limitations due to leg pain stratified by sex. In addition, we explored potential sex differences herein. We hypothesize that higher physical work demands and higher leg pain intensity increase the odds of experiencing pain that limits the ability to work.

## Methods

### Study design and setting

The present cross-sectional study regarding work-limiting pain and physical work demands bases upon data from the first wave of the SeniorWorkingLife study [42], which has been registered as a cohort study in ClinicalTrials.gov (Identifier: NCT03634410). SeniorWorkingLife is a comprehensive questionnaire survey assessing work environment and health among senior workers in Denmark (≥50 years) [42]. The SeniorWorkingLife baseline questionnaire survey was sent out between July and October 2018. Specific questions used in the present analysis are specified below. The reporting of the study followed the guidelines for the reporting of observational studies in epidemiology (STROBE) [43].

### Participants

Statistics Denmark drew a probability sample of a total of 30,000 Danes ≥50 years (18,000 in employment, 7000 unemployed, 3000 on voluntary early retirement, 2000 on disability pension), who were invited to participate in the questionnaire survey by a personal link via a web-based digital mailbox linked to each worker’s social security number (‘E-boks’) [42]. Subsequently, the survey data were merged with high-quality national registers provided by Statistics Denmark. We only included senior workers who confirmed on the questionnaire that they were employed at the time of the reply. The response rate for completing all questions in the questionnaire was 56% among currently employed workers, but in the present analyses, we also included those who did not fill out all the questions in the questionnaire (*n* = 11,786). Granted that not all participants filled in all survey questions, the exact number of participants for each analysis varies. Data from the SeniorWorkingLife study regarding the joint association of low-back pain and physical work demands with work limitations have previously been published [44].

### Ethical approval

Danish law permits scientific usage of questionnaire- and register data without collecting informed consent or applying for approval by ethical and scientific committees [45]. Statistics Denmark were responsible for depersonalizing and storing all data on their servers from where the researchers performed the analyses through remote access.

### Explanatory variables

#### Physical work demands

Physical work demands were assessed by the following survey-question: ‘How would you describe the physical activity level in your current job?’, with response options being 1) ‘Mostly sedentary work that is not physically demanding’, 2) ‘Mostly standing and walking work that otherwise is not physically demanding’, 3) ‘Standing or walking work with some lifting and carrying tasks’ and 4) ‘Heavy or fast work that is physically demanding’ [46, 47].

#### Leg pain

Participants were classified as having low, moderate, high, and very high leg pain intensities if they reported average pain intensities in the legs (hips, knees, feet) during the past three months at 0–2, 3–4, 5–6, and 7–10, respectively, on a scale from 0 to 10, where 0 indicated no pain and 10 indicated the worst possible pain.

### Outcome variable

We used the following modified version of the Standardised Nordic Questionnaire for Musculoskeletal Symptoms [48] to assess work-limiting musculoskeletal pain: ‘To which degree did the pain limit you in your work during the last 3 months?’. The response options were 1) to a very high degree, 2) to a high degree, 3) to some degree, 4) to a small degree and 5) not at all. As done in our previous publications [16, 44], these response options were subsequently dichotomized to 1) not at all to a small degree and 2) some degree to a very high degree.

### Covariates

We controlled for the following potential covariates: age, sex, BMI, smoking status, educational level, psychosocial work factors as well as musculoskeletal pain in the other body regions. Age (years), body mass index (BMI) (kg/m^2^), psychosocial work environment (0–100, specified below), and musculoskeletal pain during the past three months in the low-back, neck/shoulders, and arms (0–10, see above) were assessed on continuous scales, whereas sex (‘male’ or ‘female’), educational level (see below), smoking status (‘No, never’, ‘Ex-smoker’, ‘Yes, but not every day’ and ‘Yes, every day’), and physical activity during leisure (see definitions below) were categorical variables.

Leisure-time physical activity level was assessed by replying to the following question: ‘How would you describe your physical activity level during leisure for the last 12 months?’. Respondents were given the following response options 1) ‘Mostly sedentary’, 2) ‘Light exercise at least 4 h a week’, 3) ‘Sports or heavy physical activity at least 4 h per week’ and 4) ‘Training and competing regularly and several times a week’ [49]. Psychosocial work factors, i.e. influence at work and recognition from colleagues, were assessed on a continuous scale from 0 to 100 (with 100 being best) by specific questions from the comprehensive and validated Copenhagen Psychosocial Questionnaire [50]. Thus, the greater the score, the higher/better the collegial recognition and influence at work.

Highest attained educational level was drawn from the linked register: 1) Primary school or unknown, 2) High school, 3) Short-term higher education, 4) Medium-term higher education, and 5) Long-term higher education.

We controlled for these variables because previous studies have shown associations between work ability and sickness absence and these occupational and lifestyle factors, i.e., age [28, 29], sex [28, 29], smoking [28], overweight [51], leisure-time physical activity [49], MSD [7–9] and psychosocial work environment [29].

### Statistical analyses

The statistical analyses were carried out and controlled for potential covariates in the SAS statistical software for Windows (Proc Glimmix, SAS version 9.4, SAS Institute, Cary, NC). We applied two different statistical models. Statistical model 1 was adjusted for age, sex, and pain in the other body regions, whereas the fully adjusted model 2 was additionally adjusted for smoking status, body mass index (BMI), psychosocial work factors, educational level, and physical activity level during leisure. In order to make estimates representative for the population of senior workers, model-assisted statistical weights were applied in all analyses. Weights included sex, age, origin, highest completed education, occupational industry, as well as family income and type.

We performed logistic regression analyses of the associations between work-limiting pain (dichotomous outcome variable) and physical work demands (exposure variables), leg pain intensity, and the interaction between physical work demands and leg pain intensity. Due to the presence of a statistically significant interaction, the results are reported stratified by leg pain intensity. Results are presented as odds ratios (OR) and 95% confidence intervals (CI) unless otherwise stated, with alpha levels below 0.05 considered statistically significant differences.

## Results

The baseline characteristics of the study sample are provided in Table 1. The mean age of the respondents was 56.6 years, with around 47, 24, 23, and 6% in occupations characterized by ‘Mostly sedentary work that is not physically demanding’, ‘Mostly standing and walking work that otherwise is not physically demanding’, ‘Standing or walking work with some lifting and carrying tasks’ and ‘Heavy or fast work that is physically demanding’, respectively. Approximately 35, 37, 24, and 36% of the respondents had experienced musculoskeletal pain in the low-back, neck-shoulders, arms, or legs during the last three months, respectively, with average pain intensites of 2.8, 2.9, 1.9, and 2.8, respectively. More specifically, 11% of the entire sample reported doctor-diagnosed osteoarthritis. Overall, around 18% reported work-limitations due to pain (in all four body regions) to some, a large or very large degree, whereas the remaining 82% were either not at all or only to a small degree work-limited due to pain.

**Table 1:** Demographics and lifestyle characteristics of the included senior workers

	*n*	Mean (95% CI)	SD	% (95% CI)
Age (years)	12,879	56.6 (56.5–56.7)	5.4	
Sex				
Men	7054			53.4 (52.4–54.3)
Women	5825			46.6 (45.7–47.6)
BMI		26.4 (26.3–26.5)	5.1	
Smoking				
No, never	5714			48.3 (47.3–49.3)
Ex-smoker	4110			34.3 (33.3–35.2)
Yes, but not every day	373			3.3 (2.9–3.6)
Yes, every day	1729			14.2 (13.5–14.9)
Physical activity during leisure				
Mostly sedentary	1779			14.8 (14.0–15.5)
Light exercise at least 4 h	7202			60.9 (59.9–61.9)
Sports or heavy physical activity at least 4 h per week	2697			22.3 (21.5–23.1)
Training and competing regularly and several times a week	233			2.0 (1.7–2.3)
Psychosocial work factors (0–100)				
Recognition from colleagues	12,111	77.0 (76.6–77.4)	22.5	
Influence at work	12,128	77.5 (77.1–77.9)	23.8	
Physical activity at work				
Mostly sedentary work that is not physically demanding	5909			47.4 (46.3–48.4)
Mostly standing and walking work that otherwise is not physically demanding	2698			23.6 (22.7–24.4)
Standing or walking work with some lifting- and carrying tasks	2779			22.9 (22.0–23.8)
Heavy or fast work that is physically demanding	787			6.2 (5.7–6.7)
Work-limiting pain				
To a very large degree	166			1.2 (1.0–1.5)
To a large degree	337			3.0 (2.7–3.4)
To some degree	1578			13.3 (12.5–14.0)
To a small degree	3420			29.5 (28.5–30.4)
Not at all	6285			53.0 (52.0–54.0)

*BMI* body mass index (kg/m^2^); *n* number; *SD* standard deviation; *%* percentage

### Physical work demands and work-limiting pain

**Fig. 1:**
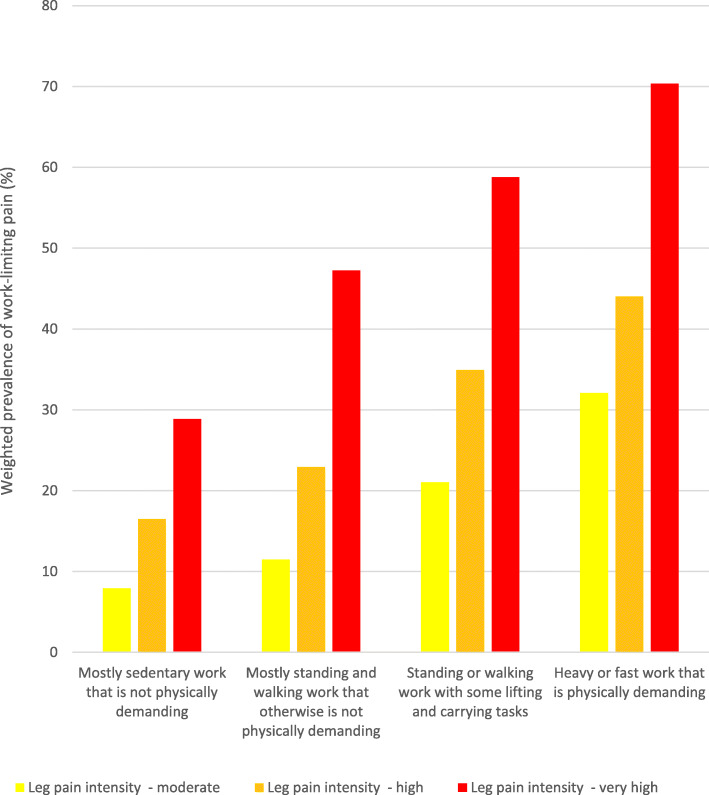
Weighted prevalences (%) of work-limiting pain among senior workers stratified by physical work demands and leg pain intensity

The weighted prevalences of work-limiting pain by physical work demands and leg pain intensity among the whole sample are provided in Fig. 1, while the associations between physical work demands and work-limiting pain stratified by sex and leg pain intensity are presented in Table 2.

**Table 2:** Odds ratios (OR) and 95% confidence intervals (95% CI) for physical work demands and work limitations due to pain stratified by leg pain intensity among senior workers

		Whole sample (women+men)				Women	Men
Pain intensity	**Physical work demands**	n	%	Model 1	Model 2	Model 2	Model 2
Moderate pain (3–4)	Standing/walking work	60	14	1.66 (1.58–1.76)	1.66 (1.57–1.75)	1.66 (1.54–1.80)	1.68 (1.56–1.82)
	Standing/walking work + lifting	105	22	2.99 (2.85–3.13)	2.71 (2.59–2.84)	2.72 (2.54–2.91)	2.69 (2.52–2.88)
	Heavy or fast work	46	37	3.82 (3.58–4.08)	3.53 (3.29–3.77)	5.49 (4.95–6.09)	2.64 (2.41–2.90)
High pain (5–6)	Standing/walking work	83	23	1.51 (1.45–1.58)	1.48 (1.41–1.55)	1.27 (1.18–1.36)	1.66 (1.56–1.77)
	Standing/walking work + lifting	173	36	2.11 (2.02–2.19)	1.97 (1.89–2.05)	2.20 (2.08–2.34)	1.80 (1.70–1.91)
	Heavy or fast work	64	44	3.29 (3.10–3.49)	3.05 (2.87–3.24)	3.20 (2.90–3.52)	2.96 (2.73–3.21)
Very high pain (7–10)	Standing/walking work	170	48	2.51 (2.41–2.61)	2.33 (2.24–2.43)	2.82 (2.66–2.98)	2.02 (1.90–2.14)
	Standing/walking work + lifting	342	60	2.97 (2.87–3.09)	2.77 (2.67–2.88)	3.64 (3.45–3.84)	2.25 (2.13–2.38)
	Heavy or fast work	180	68	4.18 (3.98–4.39)	3.84 (3.65–4.04)	5.63 (5.20–6.10)	2.97 (2.77–3.18)

The reference is ‘Mostly sedentary work that is not physically demanding’. Model 1 was adjusted for age, sex and pain in the other body regions, whereas model 2 was additionally adjusted for smoking status, body mass index (BMI), psychosocial work environment, educational level and physical activity level during leisure. Standing/walking work: Mostly standing and walking work that otherwise is not physically demanding. Standing/walking work + lifting: Standing or walking work with some lifting and carrying tasks. Heavy or fast work: Heavy or fast work that is physically demanding

Overall, we observed a significant dose-response association (*p* < 0.0001) between leg pain intensity and work-limiting pain among senior workers. We also observed a significant exposure-response association between physical work demands and work-limiting pain (p < 0.0001). In addition, a significant interaction (*p* < 0.001) between pain intensity and physical work demands for work-limiting pain existed, and the results are therefore stratified by leg pain intensity.

As it appears, the weighted prevalences of work-limiting pain generally increase with physical work demands and leg pain intensity, with a weighted prevalence of approximately 68% among senior workers with very high leg pain intensity (≥7/10) in occupations characterized by ‘heavy or fast work that is physically demanding’ (Fig. 1). As an example based on statistical model 1, the odds of work-limiting pain was significantly increased among senior workers with ‘mostly standing and walking work that otherwise is not physically demanding’ and moderate leg pain (pain intensity: 3–4/10) compared to senior workers having ‘mostly sedentary work that is not physically demanding’ (OR: 1.66, 95% CI: 1.58–1.76), and even higher odds among senior workers with ‘heavy or fast work that is physically demanding’ and moderate leg pain (OR: 3.82, 95% CI: 3.58–4.08). While the sex-stratified odds ratios follow the same aforementioned pattern (increased leg pain intensity and physical work demands being associated with increased odds of work-limiting pain) between sexes, women seem at higher odds for work-limitations due to pain compared to men, especially women with heavy or fast work.

## Discussion

The present study confirms that physical work demands and pain intensity are important factors to consider in terms of the practical consequences of MSD among senior workers, and particularly among women. In support of our hypothesis, we found a dose-response association of both physical activity during work and leg pain intensity with work limitations due to pain. These findings may call for a differentiated approach in the pursuit of sustainable employment through aging.

### Leg pain prevalence and intensity and work limitations

Low-back and neck-shoulder pain remain highly prevalent and debilitating pain regions globally [1] and have accordingly received much scientific and work environmental rehabilitative attention [3]. However, in our study among senior workers, we found equally high prevalences of musculoskeletal pain in the low-back, neck-shoulders, and legs of 35, 37, and 36%, respectively. Of the entire sample, 11% reported doctor-diagnosed osteoarthritis, which may explain some of the high leg pain prevalence, although not all cases. Overall, this underscores that the societal impact of lower body leg pain – specifically among senior workers – should not be underrated, especially not considering that lower body knee pain, specifically, has been associated with long-term sickness absence at a proportionately lower pain intensity threshold than low-back and neck-shoulder pain [14].

For the entire study sample of senior workers, we found a significant dose-response association (*p* < 0.0001) between leg pain intensity and work-limiting pain. Our results thus elaborate on previous findings demonstrating the importance of not just the presence of pain but also the *severity* of pain and functional limitation for long-term sickness absence and the ability to work [8, 9, 14, 40]. For instance, we have previously demonstrated strong dose-response associations of increasing pain intensities in the low-back, neck-shoulders, and knees with increased risk of long-term sickness absence in healthcare workers [14]. In accordance, Hallman and colleagues reported that sick leave increased and work ability decreased across all longitudinal trajectory classes of neck-shoulder pain (i.e. from low, moderate, strong fluctuating to severe persistent pain) [9]. From a rehabilitation perspective, it seems that even a small decrease in leg pain intensity could lead to a parallel decrease in work limitation due to pain. This indicates that senior workers do not have to be pain-free in order to reduce work limitations due to pain. Previous research has shown that strength training at the workplace is effective for reducing pain intensity [3, 52]. Even though strength training does not completely eliminate the pain, it reduces pain intensity with moderate to large effect sizes, making it a potent tool for workplace rehabilitation of senior workers with work limitations due to pain.

### Physical work demands and work-limiting pain

In the present study, we also demonstrated a significant exposure-response association between physical work demands and work-limiting pain among senior workers (*p* < 0.0001). This finding is in accordance with previous studies reporting a higher risk of low work ability [10, 29, 37] as well as sickness absence [33–36, 53] and disability pension [54] among workers with MSD and high physical work demands, which further emphasizes the practical consequences of MSD.

However, and importantly, our analysis demonstrated a strong significant interaction between physical work demands (*p* < 0.001) and pain intensity, indicating a combined and interacting effect of physical work demands *and* leg pain intensity on work-limiting pain.

Several studies have reported compromised work ability and productivity (presenteeism) among workers with MSD having manual jobs characterized by higher physical work demands as compared to workers with MSD in more sedentary jobs [10, 29, 37–40]. A Dutch study reported that high physical workload (including high exposures of heavy lifting, awkward and static postures) compared to low physical workload was associated with low work ability among workers with physician-diagnosed MSD [37], whereas Pensola and coworkers demonstrated that having jobs with predominantly ‘strenuous’ work tasks was associated with lower work ability compared to jobs with ‘moderately heavy’ or ‘light’ work tasks among workers with multi-site pain (including lower extremity pain) [29]. More specifically, semi-manual and manual labour was found to be significant risk factors for work-limitations (presenteeism) among workers with chronic knee pain [39]. In opposition, Wilkie and colleagues did not report manual labour as a risk factor for developing work restriction among older workers (50–59 years) with lower body MSD [40].

With regards to sickness absence, manual labour has been associated with an increased risk of sickness absence among workers with MSD [33–36]. Haukka demonstrated that having a physically light job with no exposure to lifting, carrying, kneeling, squatting and other physical work demands was associated with a lower risk of sickness absence among Finns with multi-site MSD [33]. More specifically, lower body pain due to hip and especially knee osteoarthritis has been associated with an increased risk of sickness absence in manual workers compared to non-manual workers [35, 36]. Thus, these studies together with our data could indicate larger practical consequences of having MSD among manual workers than among sedentary workers, which should call for differentiated action between working groups.

### Sex differences

While our sex-stratified analyses show similar patterns between sexes, women generally seem at higher odds of work-limiting pain. At a similar pain intensity, women with high physical work demands (heavy or fast work) seem at particularly high risk of work-limitiations due to pain (ORs of 5.49, 3.20, and 5.63 in women with moderate, high, and very high pain, respectively) compared to men with equally high physical work demands (ORs of 2.64, 2.96, 2.97, respectively). High physical work demands (by job title) have previously been associated with increased risk of sick leave and disability pension among both women and men with knee osteoarthritis [35]. As pertains to sex, arthritis-attributable work limitations have been reported to be more prevalent among women than men [55]. Likewise, sick leave and disability pension are also more prevelant among women with knee osteoarthritis than men with knee osteoarthritis [56]. Further, in a previous study pertaining work factors facilitating working beyond state pension age, women tended to be more affected by high physical work demands [57]. In opposition, the female gender (as compared to male) has been associated with good work ability among workers with multi-site pain [29], whereas other studies have reported no gender differences in the odds for work restrictions, absenteeism and presenteeism as well as risk of sick leave among workers with lower limb joint pain, chronic knee pain and knee and osteoarthritis, respectively [36, 39, 40].

Thus, both reducing the physical work demands and pain intensity may be targets that are worth pursuing by work environment professionals. As mentioned above, workplace-based exercise in terms of strength training is a well-documented beneficial means to reduce MSD pain intensity [2, 45] that may be particularly effective in terms of reducing work-limitation among senior workers with manual jobs.

Another viable strategy may be tailoring the physical work demands (e.g. reducing) to the capacities and age of the worker with MSD [58]. As example, Haukka reported that the possibility to sufficiently adjust the length of the working day was a protective factor for sickness absence in workers with MSD [33]. In the Nordic countries, however, it currently seems that older manual workers experience similar or even higher occupational physical demands compared to younger workers [58, 59]. In addition, we have previously reported a strong labour market inequality in opportunities offered by the workplace for supporting a long and healthy worklife, whereby senior workers in manual labour are offered fewer opportunities such as exercise training during work compared to sedentary senior workers [60]. This poses a further threat to prolonging worklife among senior manual workers [47] and needs to be further addressed in future studies.

### Strengths and limitations

The present study contains several strengths and limitations. It is a clear strength of the study that the invitation of potential participants was based on a probability sample drawn by Statistics Denmark among all eligible Danish residents ≥50 years. Further, all analyses were performed by employing statistical weights based on national registers, which ensured that the data was representative of senior workers in Denmark, and reduced the potential influence of non-response bias. The large and representative sample strengthens the statistical power and reduces the chances of statistical type II errors. A limitation of the study is the cross-sectional design, which does not allow for causal inferences and entails the risk of reverse causation.

The step-wise adjustment for potential covariates in the statistical models only changed the odds-estimates to a very small extent. Thus, it seems that educational attainment, physical exercise, smoking, BMI, and psychosocial work factors are less important factors for the interplay between pain intensity and work-limiting pain. However, other studies show that both overweight and smoking may be work-limiting in terms of increasing the risk of sickness absence [61].

The presented results may also be biased by ‘the healthy worker effect’ as we only included senior workers still capable of working and excluded those that have left the labor market prematurely or changed to a less physically demanding job due to health problems such as MSD. Thus, it cannot be ruled out that individuals with the longest history of physically and mentally demanding work could already be outside the labour market or in another job when the SeniorWorkingLife study was initiated. Hence, we consider our odds estimates to be relatively conservative and may thus underestimate the actual odds estimates. We used self-reports to determine physical activity during work, which may have introduced self-report bias and inaccuracy [62], and hence represent a limitation to the study. However, we have previously demonstrated strong agreement between the specific research item used regarding physical work characteristics and grouping based on ISCO (International Standard Classification of Occupations 1), where respondents are stratified into occupational groups based on high-quality national registers [47]. Additionally, self-reporting can result in common method variance where the answers may be influenced by e.g. the respondents mood, health status and socioeconomic status [63]. Furthermore, different time frames between variables (leg pain intensity, physical activity in leisure, current physical work characteristics etc) also constitute a limitation of the study. Future studies investigating the joint association of physical work demands and pain intensity with work-limiting pain may employ other methods such as technical measurements of physical activity during a working day or using job titles.

## Conclusion

Physical work demands and pain intensity are important factors to consider with regards to the practical consequences of MSD among both female and male senior workers. Here, we report dose-response associations of both physical activity during work and leg pain intensity with work limitations due to pain, with stronger associations noted in women than men. These findings suggest that approaches in the pursuit of sustainable employment through aging should be differentiated between work groups and sexes.

## Data Availability

The authors encourage collaboration and use of the data by other researchers. Data are stored on the server of Statistics Denmark, and researchers interested in using the data for scientific purposes should contact the project leader Prof. Lars L. Andersen, lla@nfa.dk, who is responsible for the study design, questionnaire development, definition of population, and data collection.

## Abbreviations

95% CI: 95% confidence intervals
BMI: Body mass index
OR: Odds ratio
MSD: Musculoskeletal disorders
LTSA: Long-term sickness absence
